# Success and opportunities of the American Academy of Pediatrics Marshall Klaus research grant program in neonatal-perinatal medicine

**DOI:** 10.1038/s41372-024-02137-5

**Published:** 2024-10-05

**Authors:** Albertina Lee, Joern-Hendrik Weitkamp, Angie Tune, Jim Couto, Krithika Lingappan

**Affiliations:** 1 Division of Neonatology and Department of Pediatrics; Children’s Hospital of Philadelphia and University of Pennsylvania, Philadelphia, PA USA; 2 Mildred Stahlman Division of Neonatology, Department of Pediatrics, Monroe Carell Jr. Children’s Hospital at Vanderbilt, Vanderbilt University Medical Center, Nashville, TN USA; 3 Hospital & Surgical Subspecialties, American Academy of Pediatrics, Itasca, IL USA

**Keywords:** Scientific community, Ethics

## Abstract

**Background:**

Physician-scientists are a crucial link between clinical practice and research. The American Academy of Pediatrics (AAP) initiated the Marshall Klaus Perinatal Research Award to enhance the development of research skills among physicians training in Neonatal-Perinatal Medicine.

**Methods:**

In this study, we sought to identify trends in funding along with geographical and demographic variables of the applicants and mentees and assess the applicants’ scholarly productivity and funding from the National Institutes of Health (NIH). We reviewed the data of applicants and awardees from 2015–2024.

**Results:**

We found that basic science applications had a higher funding likelihood than clinical/translational applications. The geographical distribution of awardees is skewed. There was a significant association between awardee status and K08 or K23 funding attainment.

**Conclusions:**

Future efforts should support more equitable award distribution and a diverse research landscape in neonatal-perinatal medicine.

## Introduction

Physician-scientists play a pivotal role in the biomedical workforce as a crucial link between clinical practice and research. Their unique position allows them to translate clinical observations into research questions and vice versa. However, the sustainability of this career path is under threat due to several challenges. These include a decline in interest in research careers among graduating medical students, increased age at first R01 grant success for physicians, and fewer physicians reporting research as their primary work activity. Furthermore, there is a significant gender and racial disparity within the physician-scientist workforce, with women and underrepresented minorities facing barriers in advancing their careers [1–3].

Early funding for physician-scientists is essential to maintain and grow the physician-scientist workforce. Strategic funding programs are necessary to provide education in research methodology, generate data from pilot experiments, and increase competitiveness for extramural funding from the NIH [4–7]. These measures can support the clinician-scientists whose scientific questions often arise at the bedside and are critical for advancing patient care. The American Academy of Pediatrics (AAP), Section on Neonatal-Perinatal Medicine (SoNPM), initiated the Marshall Klaus Perinatal Research Awards to enhance and support the development of research skills among physicians training in Neonatal-Perinatal Medicine. These awards provide financial support to assist outstanding fellows in initiating or completing their research projects. Marshall Klaus awards have been funded by the AAP/SoNPM (2015–2024), March of Dimes (2015), an endowment from Johnson & Johnson Pediatric Institute (2015–2024) and unrestricted industry grants from Prolacta (2018–2020) and Reckitt/Mead-Johnson Nutrition (2016–2023).

The Marshall Klaus Perinatal Research Awards, created and awarded by the section of Neonatal-Perinatal medicine, have been in existence since 2005 in memory of the late Marshall H. Klaus, M.D., and is a testament to his significant contributions to the field of neonatology. Dr. Klaus, an internationally renowned neonatologist and scientist, authored several seminal works, including maternal and parent-infant bonding books. His teachings at the University of California, San Francisco, School of Medicine, and his impactful research have left an indelible mark on American medical schools and beyond. The research grants are awarded to fellowship trainees in Neonatal-Perinatal medicine based on the merit of their application.

Projects are related to the full spectrum of child health research, such as perinatal-neonatal health, behavioral sciences, biomedical science, epidemiology, health services, prevention, public health. The expectation is that trainees must conduct their research under the supervision of a mentor within their department or with other basic, clinical, or social scientists within the trainee’s institution and should be completed during fellowship. Since 2015, applications were submitted electronically through a dedicated AAP SoNPM web portal (https://www.aap.org/en/community/aap-sections/sonpm/marshall-klaus-perinatal-research-awards/). Announcements for applications have been relatively consistent and have included emails to members of the AAP section of neonatal-perinatal medicine, emails to perinatal-neonatal medicine fellowship program directors, announcements at the annual AAP NCE meeting, and AAP SoNPM-sponsored social media.

In 2015, we performed a survey among 41 awardees to assess the impact of the Klaus Award on their academic careers in neonatology [8]. Of the responders, almost 2/3 worked at

academic institutions with more than half having academic appointments. In this study, we reviewed all applications since 2015 sought to identify trends in funding and research applications, the geographical distribution of applicants and awardees, and the demographic variables of the applicants and mentees. Lastly, we sought to assess the applicants’ scholarly productivity and funding from the National Institutes of Health (NIH).

## Methods

We reviewed the data of Marshall Klaus applicants and awardees from 2015 to 2024. Demographic data of applicants and mentors was extracted from this database. Data was reported as a mean for continuous data and median for non-continuous variables. The authors designated the research application as basic or clinical/translational based on the research title and/or the methods included in the abstract of the published manuscript (in PubMed) with the same title as the title of the Marshall-Klaus research application. The research was classified as “clinical/translational” if any human subjects or human samples were used in the study and as basic science if the project was based on pre-clinical models exclusively. This classification was blindly assigned by two researchers who agreed on the designation.

The manuscript did not include awards under the health services research and education research because the review process and criteria for these two awards were not the same as for the Clinical/Basic Science research proposals. The Beth Israel Deaconess Medical Center sponsors the Health Services Award, while the Education Research Award is sponsored by Brodsky & Martin’s *Neonatology Review s*eries.

Clinical/Basic Science research applicants were asked to submit (1) Project title, (2) Specific aims, (3) Research strategy, (4) Reference, Budget, and Timeline, (5) Applicant Biosketch, (6) Mentor Biosketch, (7) Mentor Letter, and (8) Letter from the Fellowship Program Director.

After the applications are received, an independent panel of three reviewers not affiliated with the applicant’s institution score each application based on the following criteria: significance of the topic, innovation, proposed methodology, feasibility to complete during the fellowship, and relevance to the trainee’s career goals. Each application is reviewed by these three non-conflicted reviewers, and in addition to scoring, each reviewer is asked to rank the applications. Rankings are averaged among all three reviews to determine the final ranking of all submitted applications. If one review is a significant outlier, the committee chair assesses the application and determines the final ranking. Reviewers with conflicts recuse themselves where a conflict arises (same institution, having published with a mentor, having trained a mentee now at a different institution).

Reviewers were recruited with established expertise in e clinical and/or basic science expertise as documented by their publications, grants, and research-related positions in academic institutions. The reviewers are appointed by the research chair (term limit of a maximum of 10 years). The goal has been to include reviewers representing most, if not all districts. There is a mix of gender, and both junior and senior faculty members are represented and to appoint reviewers with active or a history of NIH grant funding.

Publications were identified and quantified by searching for author’s name in PubMed. NIH grants were identified and quantified by searching for the applicant’s name in NIH-Reporter.

Welch’s *t* test was used for the comparison of the percentage of category-specific grants funded. Fisher’s exact test was used for the statistical analysis of K23 versus K08 awards among awardees and non-awardees. Statistical analysis was done using GraphPad Prism Version 10. *P*-value of <0.05 was considered significant.

## Results

### Basic science applications had a higher likelihood of funding compared to clinical/translational applications

**Fig. 1:**
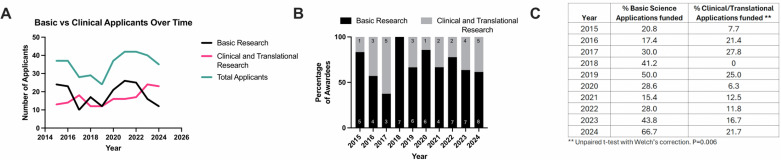
Basic science applications had a higher likelihood of funding compared to clinical/translational research applications. **A** Trend in the number of applicants submitting basic research or clinical/translational research applications from 2015–2024. **B** Basic vs clinical/translational research composition of projects funded by the Marshall Klaus award by year. Results are shown as percentages and individual research type categories labeled with sample size within the bar. **C** Percentage of projects funded/number of category-specific applications by year. Results are shown as percentages and individual research-type categories are shown. Statistical comparison of the percentage of category-specific grants funded between basic research and clinical/translational research across all years was made using unpaired t-test with Welch’s correction. *P* = 0.006. The unpaired t-test with Welch’s correction was chosen as it does not assume the two populations have the same standard deviation. The variances were not different between the groups. The difference between the means (translational/clinical- basic) ±SEM was −19.10 ± 5.841, 95% confidence interval was −31.62 to −6.573.

Research with preclinical models was classified as “Basic Science,” while research involving human subjects (clinical trials or translational studies involving human samples) was classified as “Clinical/Translational.” For this study, we only included research applications categorized as “Basic Science” or “Clinical/Translational”. From 2015 through 2024, we identified varying trends in the total number of applications received per year and the proportion of basic and clinical science applications (Fig. 1A). The number (%) of basic and clinical/translational applications received each year is shown in Supplemental Table 1. The number of awards granted varied yearly depending on available funding, from a minimum of 6 to a maximum of 13 in 2024. The percentage of basic science awards was calculated from the total basic science applications received for a particular year, and the same approach was followed for the percentage of clinical awards. However, basic research applications were significantly more likely to be funded than clinical and translational applications. Evaluating the percentage of category-specific grants funded yielded a significant difference (*p*-value = 0.006 < 0.05) between the percentage of total basic research grants funded compared to total clinical or translational research grants for the entire period evaluated (Fig. 1C).

### Single-degree applicants (MD or DO) comprised the majority of awardees compared to those with additional degrees

**Fig. 2:**
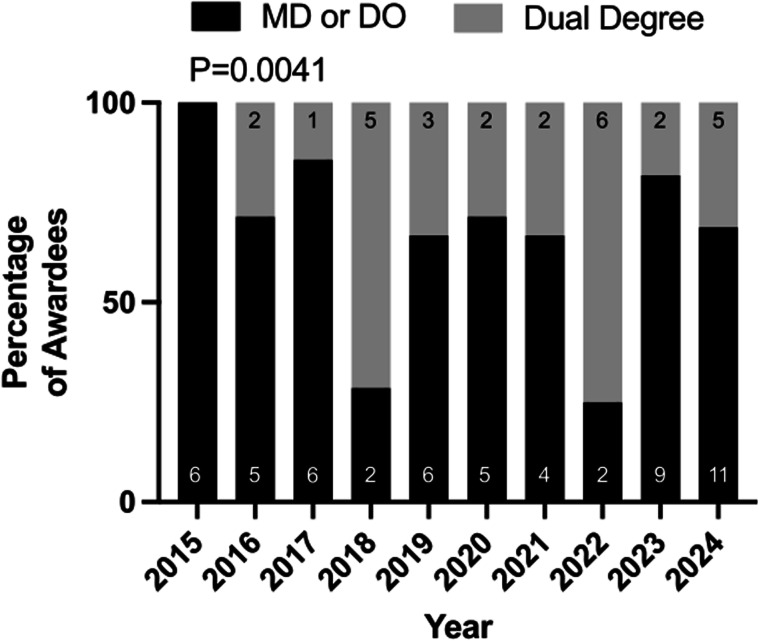
Degree distribution of awardees selected. Categories reported as MD or DO and dual degrees (MD-PhD, MD-MS, MD-MPH). International medical degrees excluded. Results reported as percentage of awardee pool. Individual degree subcategories labeled with sample size on bars.

We evaluated the variation in degrees (single vs. dual degree) among awardees. We divided them into two categories: single medical degrees (Doctor of Medicine (MD) or Doctor of Osteopathic Medicine (DO)) and dual degrees (MD-PhD, MD-MS, MD-MPH). Apart from two years (2018 and 2022), the awardee pool had a majority of MD/DO trainees (Fig. 2). In the evaluation period, awardees with dual degrees were mostly MD/PhD candidates. One recipient with an international medical degree was excluded from the comparison. Notably, the degrees of all applicants were not available for this analysis. Degrees were available for awardees only.

### The geographical distribution of awardees is skewed

**Fig. 3:**
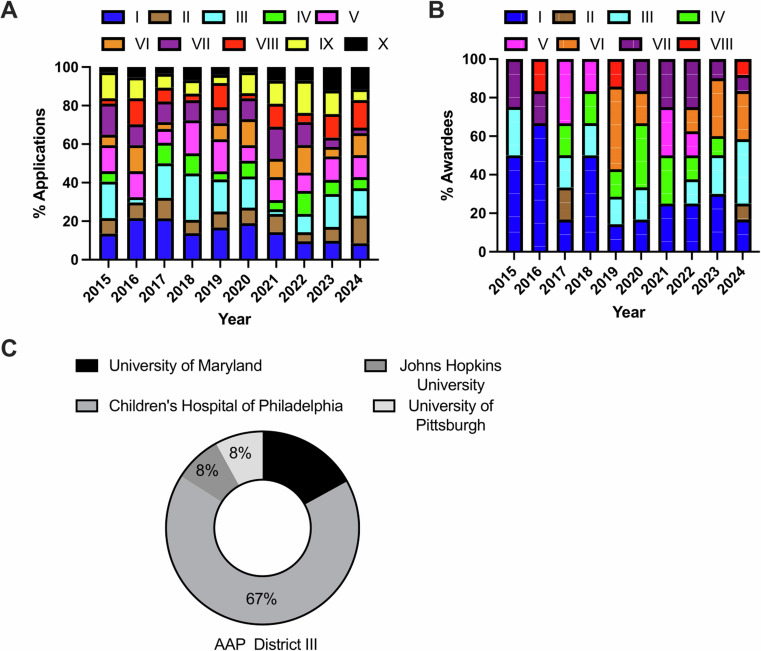
The geographical distribution of awardees is skewed. **A** American Academy of Pediatrics (AAP) district distribution of applicant fellowship institutions by year. Results are reported as a percentage of the yearly total. **B** AAP district distribution of awardee fellowship institutions by year. Results are reported as a percentage of the yearly total. **C** Fellowship institution composition of all applications awarded from AAP District III. Results are reported as a percentage of all applications funded from District III.

AAP Districts I to X were all represented annually in application affiliation profiles (Fig. 3A). The distribution of states within each AAP district in included in Supplementary Table 2. However, this diversity was not reflected in the profiles of those who were selected for the award, such as Districts IX and X so far did not yield successful applicants (Fig. 3B). Further evaluations of the fellowship institutions dominating awardee profiles were made in AAP Districts I and III. These respective districts were the top two AAP districts that were funded by the Marshall Klaus Basic/Clinical Award over all analyzed years. Out of the total number of awards granted (*n* = 83), those awarded from District I (*n* = 20) were all affiliated with the Harvard Neonatal-Perinatal Fellowship Training Program (*n* = 18) from the Boston Children’s Hospital (BCH). On the other hand, those awarded from District III (*n* = 12) were mostly affiliated (67%) with the Neonatal-Perinatal Fellowship Training Program from the Children’s Hospital of Philadelphia (*n* = 8) (Fig. 3C).

#### Awardees had a higher trend of scholarly productivity compared to non-awardees

**Fig. 4:**
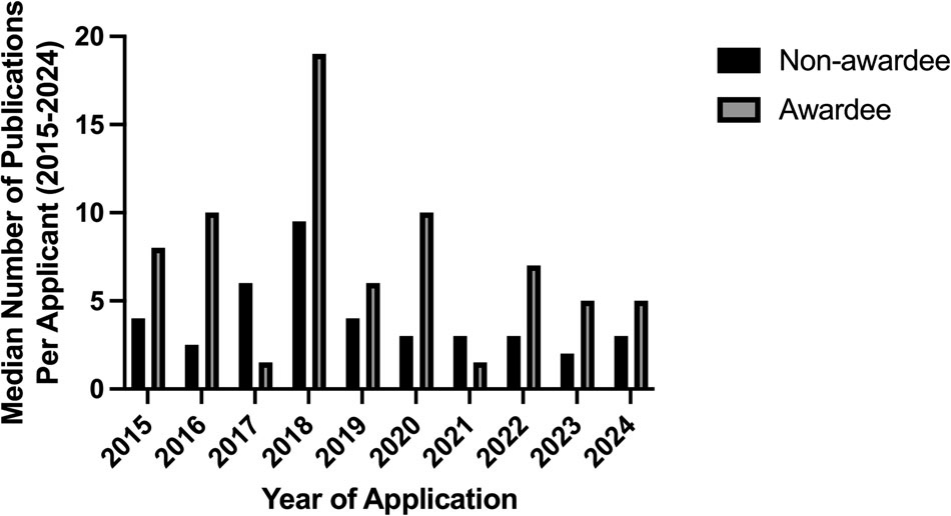
Awardees had a higher trend of scholarly productivity compared to non-awardees. Comparison of the total scientific publications after the year applied between awardees and non-awardee applicants. Results are shown as a median number of publications per applicant.

Next, we assessed the scholarly productivity of the applicant pool (Fig. 4). The applicants were categorized based on the year that they applied for Marshall Klaus award, and the number of publications from the year of application to 2024 are represented in Fig. 4. There was no significant difference in publication productivity between those selected and those not chosen for the award.

#### Awardees have significantly greater success at attaining K08 awards compared to non-awardees

**Fig. 5:**
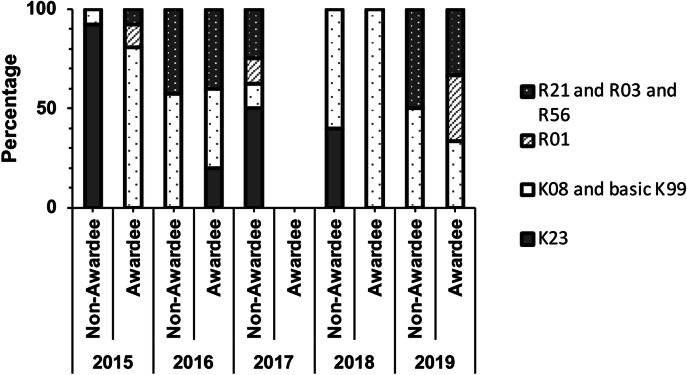
Awardees have significantly greater success at attaining K08 awards compared to non-awardees. NIH research grant awards (K- and R- series) comparison between awardees and non-awardees. Results are shown as the percentage of total awards between 2015 and 2019.

We evaluated NIH grant productivity in four categories across four years from 2015 to 2019 (Fig. 5A). We assessed NIH grant productivity from 2015 to 2019 to consider the timeline of a K-grant submission after completion of the fellowship. We segregated grant categories across four sectors: Mentored Patient-Oriented Research Career Development Award (K23) only, Clinical Investigative or Pathway to Independence Awards (K08 and K99), Research Project Grant (R01) only, and Exploratory and/or Short-Term Awards (R21 and R03 and R56).

We identified 49 K08/K99 awards in the applicant pool, out of which 11 (22%) were non-awardees and 38 (78%) were Marshall-Klaus awardees. We identified 19 K23 awards in the applicant pool, out of which 18 (95%) were awarded to non-awardees, and one grant was awarded to a Marshall-Klaus awardee. When assessed by Fisher’s Exact test, there was a significant association between awardee/non-awardee status and attainment of K08 or K23 funding (*P* < 0.0001, Odds ratio: 0.02, 95% CI for odds ratio: 0.001509–0.1091).

While there are other important non-NIH grant mechanisms that Marshall Klaus awardees are likely to pursue, such as the Basil O’Connor award from the March of Dimes, we did not have enough award information to evaluate on these factors as a basis of scholarly productivity.

## Discussion

Our analysis of the Marshall Klaus Basic/Clinical Science Awards from 2015 to 2024 reveals several key trends and disparities in funding and mentorship based on research category, gender, applicant degree type, and geographical distribution of the fellowship program. Furthermore, we evaluated the impact of the Marshall Klaus grant on scholarly productivity and NIH mentored and independent grant funding.

Firstly, our findings indicate that basic science applications, which include research involving animals and lab-based studies, are significantly more likely to be funded than clinical/translational applications involving human subjects (*p*-value = 0.006). As the evaluation criteria of the grants are uniform across all applications and reviewers with expertise in clinical or basic science, review them, selection bias towards basic science applications may not be the reason behind this finding. Going forward, a collective onboarding session of all the reviewers about the scoring criteria may lead to a more uniform understanding of the criteria and scoring. Also, attempts may be made for funding the top-scoring clinical/translational and basic science awards as separate categories by reviewers with specific expertise in the respective category.

Our examination of degree types among awardees revealed that those with single medical degrees (MD or DO) comprised the majority of awardees. Without knowing the background percentage of single versus double degree Neonatal/Perinatal fellowship trainees it is difficult to evaluate the implication of the frequency of applications from single degree versus double degree trainees.

Geographically, the distribution of awardees was skewed, with significant representation from AAP Districts I and III. District I awardees were overwhelmingly affiliated with the Harvard Neonatal-Perinatal Fellowship Training Program, while District III awardees were mostly from the Children’s Hospital of Philadelphia. This concentration of awardees from specific high-profile institutions highlights potential inequities in research funding distribution but may reflect the concentration of mentors in these institutions. In addition, imbalanced institute affiliation of committee members may also contribute to this geographical skewed distribution. We also acknowledge redundancy of mentors who have had awards funded, where we found four duplicates in mentors (out of 80) whose trainees have been funded over all the years evaluated.

Finally, our assessment of NIH grant productivity revealed that awardees had significantly greater success in attaining K08 awards than non-awardees (*P* < 0.0001). This indicates that the Marshall Klaus Award may be a valuable steppingstone for securing further NIH funding and advancing academic careers in neonatology. But it also points out gaps to fill to support more clinical/translational research among applicants. A greater number of Marshall Klaus awardees were engaged in basic science research and went on to attain the NIH K08 award. Among non-awardees, there was a higher attainment of a K23 grant.

We recognize the limitations of this study. Information regarding race and ethnicity was not collected, so we cannot assess the diversity in applications and awards by this very important variable. The gender of the applicants and the mentors were not self-reported by the mentors or the applicants at the time of application. Not all publications and their impact factors were captured, limiting the assessment of scholarly productivity between applicants. The degrees of the applicants were not available, so we were not able to assess the success rate in double-degree and single-degree applicants. Because of their longer background in research, dual-degree applicants are more competitive due to their past productivity and successful mentoring relationships with their mentors. Nevertheless, single-degree applicants were very successful in our cohort and are a significant part of the research community in neonatal-perinatal medicine. We recognize that there are other extramural award mechanisms that the Marshall Klaus awardees could have applied for, but we cannot assess those mechanisms.

In conclusion, our study highlights important trends in the Marshall Klaus perinatal research funding program. The findings inform future efforts for more equitable opportunities and a more diverse research landscape in neonatal health sciences. The recently added award mechanisms for health services research and education promote a broader spectrum of leadership and academic career opportunities in neonatology. Understanding and mitigating factors contributing to geographical and topical inequities will foster the careers of physician-scientists in neonatal-perinatal medicine in all domains.

## Supplementary information

Supplementary Table 1

Supplementary Table 2

## Data Availability

Datasets used in this study are made available to referees at submission and to readers upon request.
