# Supplementary figures and images for: Success and opportunities of the American Academy of Pediatrics Marshall Klaus research grant program in neonatal-perinatal medicine
Source: J Perinatol. 2024 Oct 5;45(5):595–9. doi: 10.1038/s41372-024-02137-5 (PMC12222013; doi:10.1038/s41372-024-02137-5)

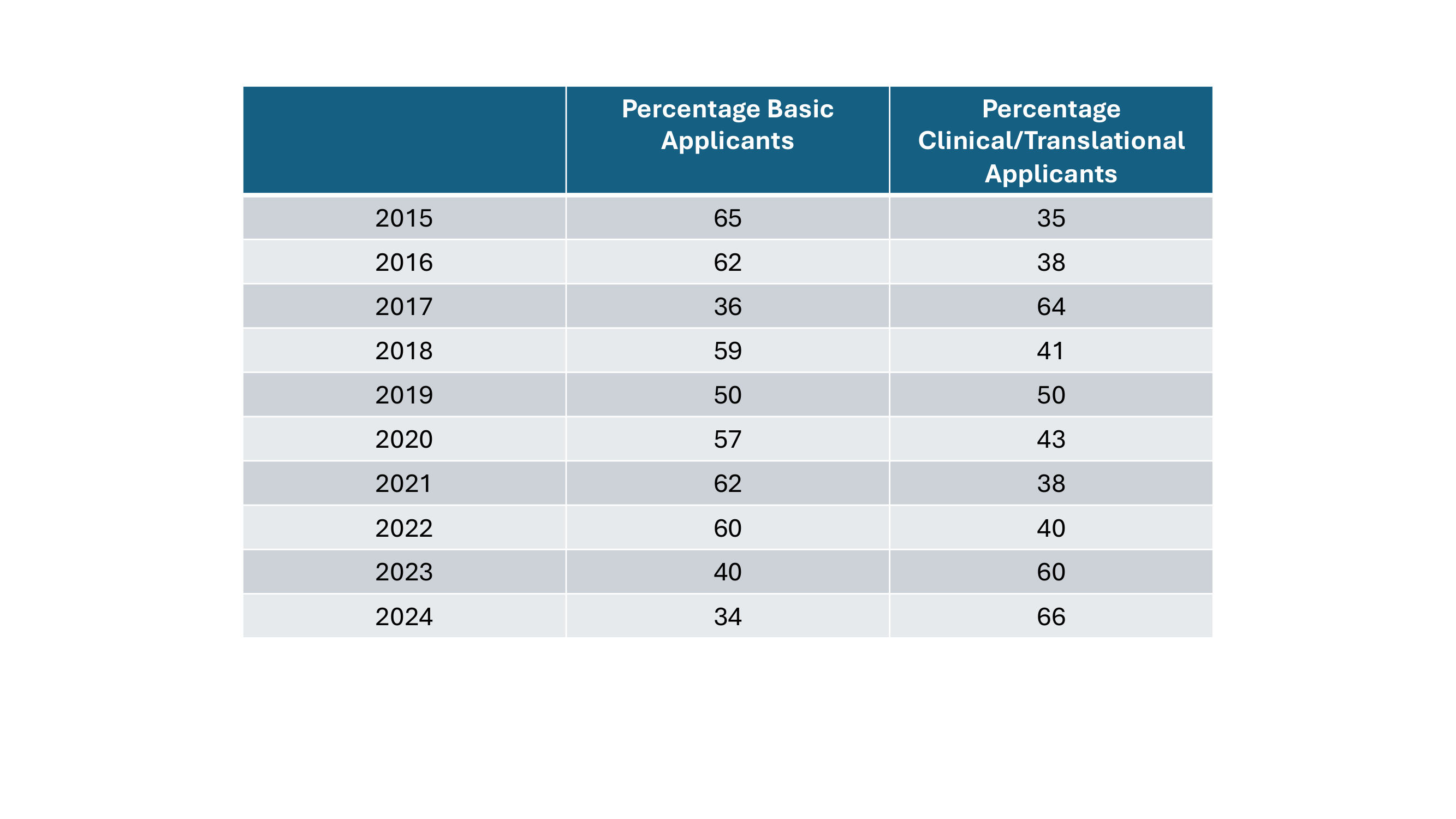

Supplement: Supplementary file 1 — Supplementary Table 1 [file 41372_2024_2137_MOESM1_ESM.tif]

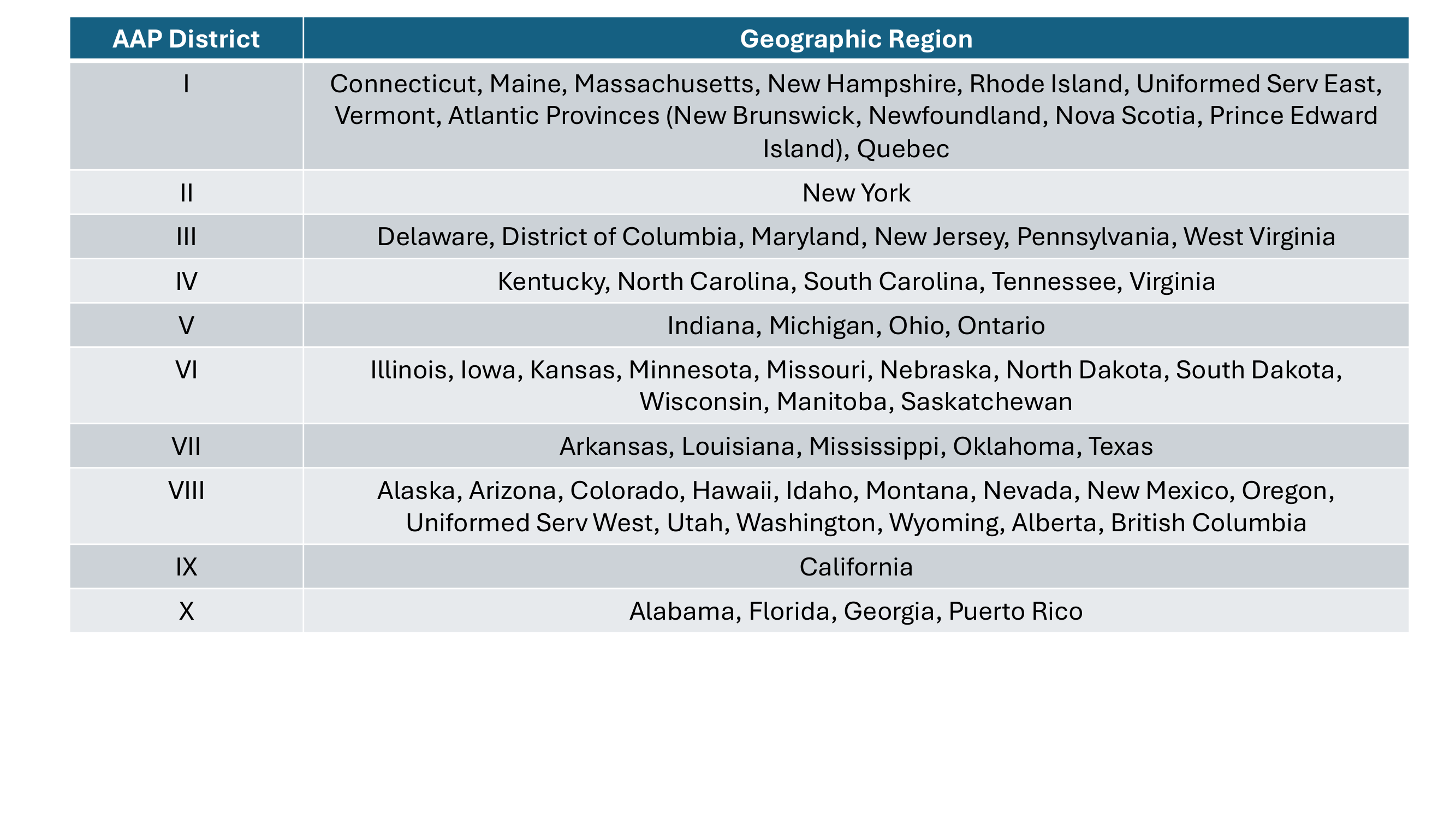

Supplement: Supplementary file 2 — Supplementary Table 2 [file 41372_2024_2137_MOESM2_ESM.tif]
